# Dataset on powered two wheelers fall and critical events detection

**DOI:** 10.1016/j.dib.2019.103828

**Published:** 2019-03-16

**Authors:** Abderrahmane Boubezoul, Fabien Dufour, Samir Bouaziz, Bruno Larnaudie, Stéphane Espié

**Affiliations:** a University Paris-Est, IFSTTAR, TS2-SIMU&MOTO, 14-20 Boulevard Newton, F-77447 Marne-la-Vallée, France; b TROPHY R&D, 1 Avenue Eiffel, 78420 Carrières-sur-Seine, France; c University Paris-Sud, SATIE Laboratory, 91405 Orsay, France

## Abstract

In this data article, we will present the data coming from 3D Inertial Measurement Unit (3-accelerometers and 3-gyroscopes sensors) mounted on the motorcycle collected during a motorcycle's falls experiments. Developing a motorcycle's fall events detection algorithms is a very challenging task because the motorcycle falling is multi-factorial and is strongly influenced by many unknown factors. To solve this issue, one solution can be to use a data-set collected during controlled experiments, knowing that the real motorcycle falls cannot be replicated, a stuntman can be chosen to be as close to reality as possible. The experiments have been conducted based on predefined scenarios such as: fall in a curve, fall on a slippery straight road section, fall with leaning of the motorcycle ‘‘intentional manoeuvre’’ and fall in a roundabout. These scenarios have been designed based on realistic falls. Other experiments have been conducted under different extreme driving situations. These extreme manoeuvres were carried out on track by professional riders. The purpose of performing these manoeuvres was to obtain a dataset describing the limit handling behaviour.

**Table:** Specifications table

Subject area	*Transportation*
More specific subject area	*Powered two wheelers safety, events*
Type of data	*Text files*
How data was acquired	*Experiments were conducted using an instrumented motorcycle rode by professional riders on tracks*
Data format	*Raw 3D Inertial Measurement Unit (3-accelerometers and 3-gyroscopes sensors) mounted on the motorcycle collected during a motorcycle's falls experiments)*
Experimental factors	Measure of dynamic behaviour of motorcycle-rider system during fall and near fall events
Experimental features	*Experiments were conducted using an instrumented motorcycle rode by professional riders on tracks*
Data source location	*Ile de France, France*
Data accessibility	Data is with this data article as supplementary material to aid reproducible research
Related research article	*A. Boubezoul, S. Espié, B. Larnaudie, S. Bouaziz “A simple fall detection algorithm for powered two wheelers”, Control Engineering Practice, 21 (3) (2013), pp. 286-297*

**Table:** 

**Value of the data** • This data collection aims to improve our knowledge about the dynamic behaviour of motorcycle-rider system during fall events, and to design a robust algorithm which could be used to trigger any alert system. • The data acquisition system was designed to capture the signature of the fall or the near-fall which is characterized by a high dynamic behaviour. • The high resolution collected data (the acquisition frequency is 1000 samples per second) is very useful for pre-fall and post-fall analysis. • In the scenario design step, the scenarios that usually represent the accident situations encountered by the riders were selected. • This data set is gathered from real controlled experiments which can be useful for the validation of the fall detection algorithms.

## 1 Data

In this data article, we present.

Data coming from the 3D Inertial Measurement Unit (combination of 3 individual accelerometer + gyroscope sensors) mounted on the motorcycle. Measurements were collected during:

• Controlled experiments on tracks performed by a stuntman (fall experiments). The fall scenarios were based on accident analysis reports and designed to reproduce as closely as possible the following situations: fall in a curve, fall on a slippery straight road section, fall with leaning of the motorcycle ‘‘intentional manoeuvre’’ and fall in a roundabout [2].
• Near-fall experiments that were carried out by professional riders. For the near-fall condition, the goal was to observe the limit of handling behaviour of the motorbike: extreme braking, accelerating manoeuvres, and fall-like manoeuvres.

Presented in this article are figures and table showing the most important steps in the fall and near-fall scenarios experimental design.

**Fig. 1:**
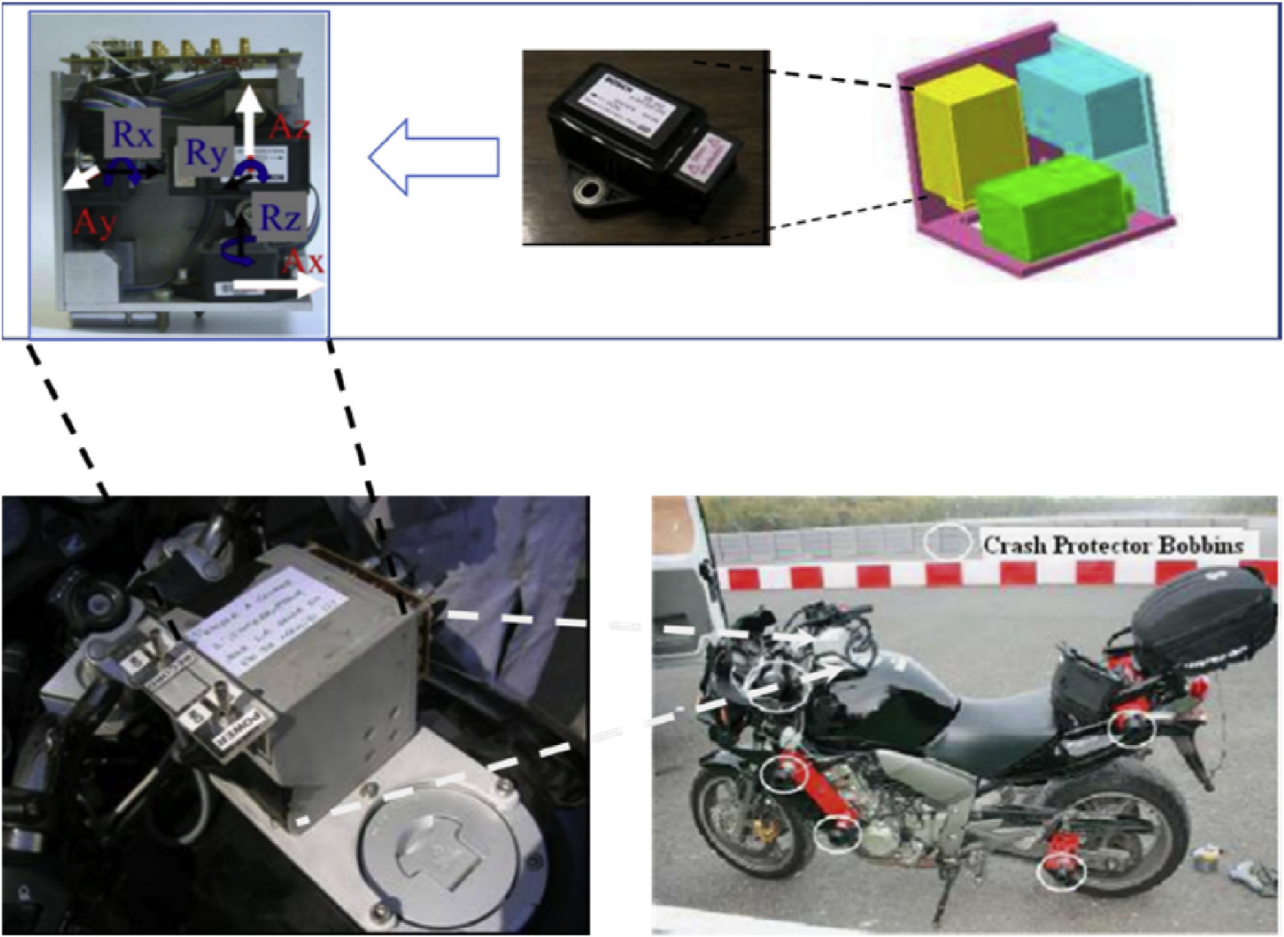
The 3D Inertial Measurement Unit (3-accelerometers and 3-gyroscopes sensors) mounted on the motorcycle. The accelerations and rotations are expressed in the reference triad.

**Fig. 2:**
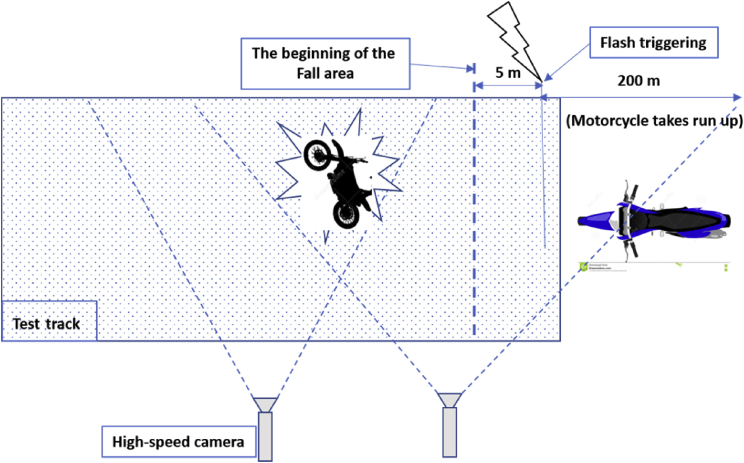
Test track where the experiments were conducted.

**Fig. 3:**
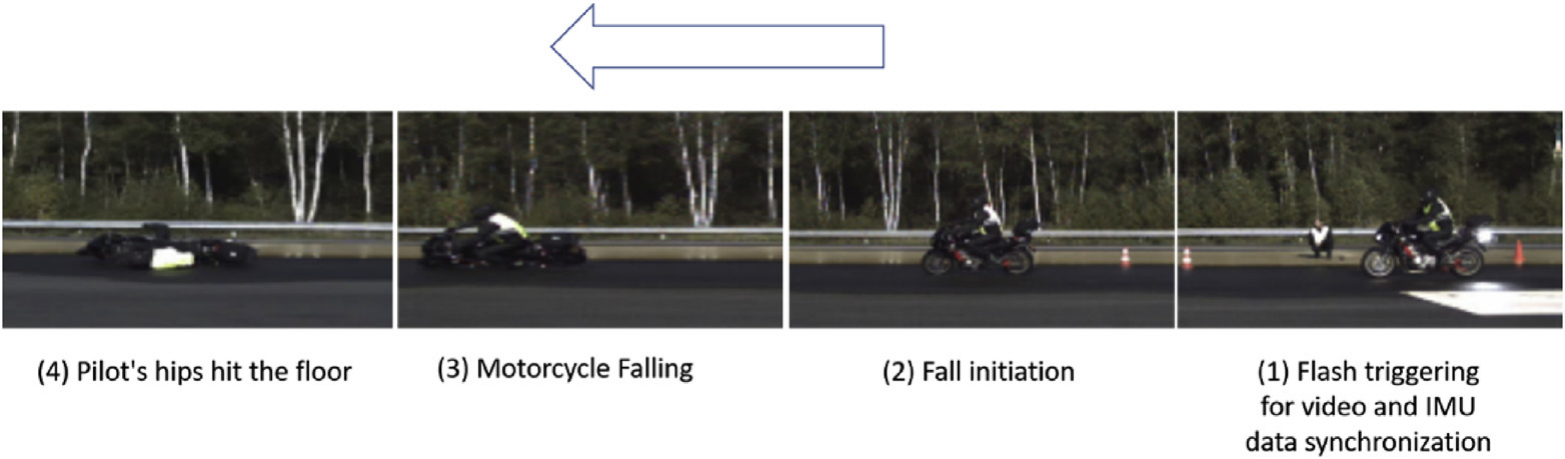
The recorded video with a high-speed camera 1000 pictures per second.

**Fig. 4:**
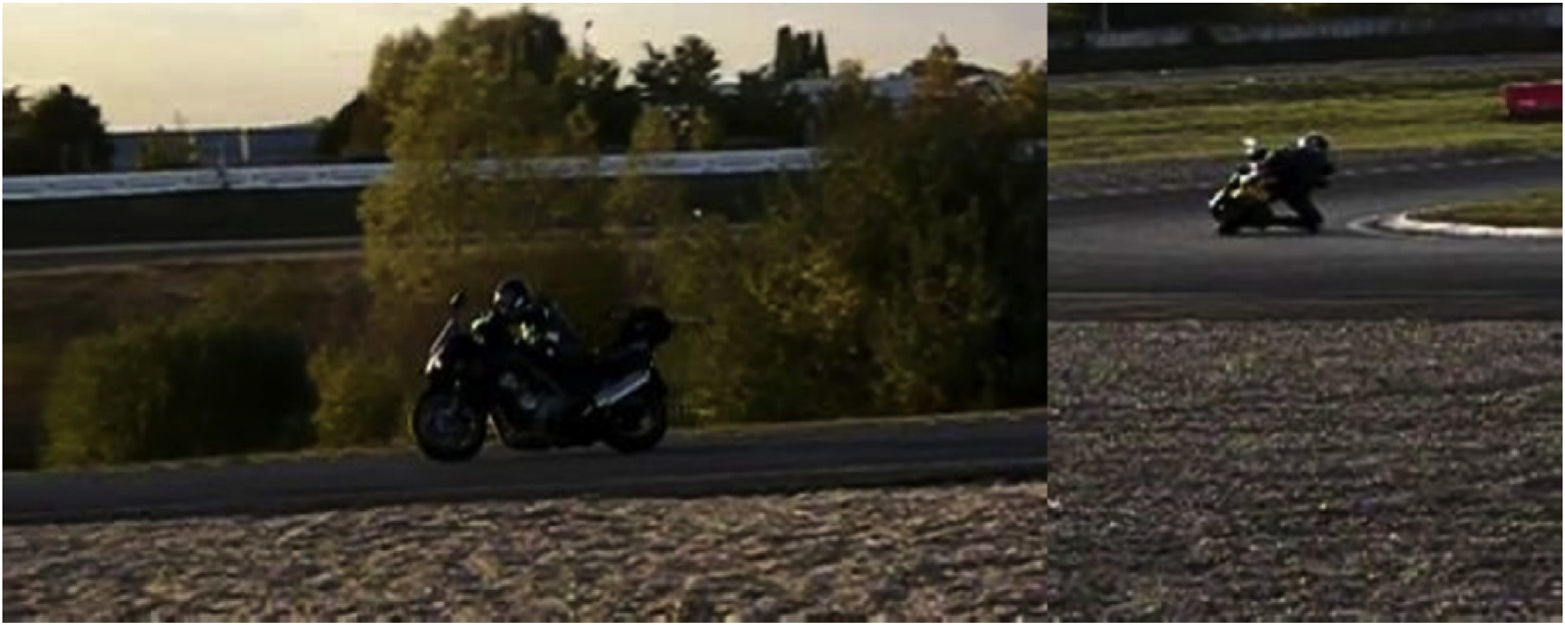
Recorded video of sporty driving situation. The professional rider is riding at high speed and giving a high lean angle to the motorcycle.

The figures presented in the following sections describe the experimental design of the fall and near-falls scenarios. Fig. 1, shows the instrumented motorcycle and the sensors installed on it. Fig. 2, displays the fall scenarios design on the track, and a video footage of a fall scenario is given on Fig. 3. Finally, Fig. 4, shows a recorded video of sporty driving situation.

Table 1, gives the fall start time and the fall time for each fall scenario.

**Table 1:** The different performed scenarios with the corresponding fall start time and the fall time for each scenario.

Scenario	The fall start time a (ms)	The fall time b (ms)
Fall on a slippery straight road section	40132	40428
Fall with leaning of the motorcycle	34288	34502
Fall in the roundabout	35876	36160
Fall in a curve	43486	43756

a This time corresponds to the time where the lowest crash protector bobbin hits the ground.

b The fall end time corresponds to the time where the stuntman's hips hit the floor.

## 2 Experimental design, materials, and methods

PTW riding is a very complex compared to driving four wheeled vehicles, due to the fact that the rider must actively maintain the dynamic stability of his/her vehicle. This function is demanding, particularly during emergency events such as in the case of harsh braking in curves. In such situations, the loss of stability may induce a fall. The detection of initiation of the PTW fall is non-trivial, due to the multi-factorial determinants. Many studies have sought to understand the fall and the factors that contribute to this kind of accident, (see MAIDS, 2009) [1]. During a fall, the PTW is subjected to high dynamic forces due, for instance, to the braking action and its intensity and when hitting the ground, to high frequency oscillations as in bending of the vehicle frame and rotations of the handlebar. For accurate detection of the fall or near-fall signature, a specific data collection system was designed to capture the sensors signals, at a 1Khz sampling rate, using a 4 μs time stamping.

The collected data come from a 3D Inertial measurement unit (accelerometers/gyroscopes). To obtain the linear accelerations (lateral, longitudinal, vertical) and angular velocities (roll, yaw, pitch) of the motorcycle we used a BOSCH automotive sensor, the gyroscope sensor has a 100°/s resolution and the Coriolis acceleration in ±1.8 g, see Fig. 1.

## 3 Fall scenarios design

The experiments were conducted based on predefined scenarios such as: fall in a curve, fall on a slippery straight road section, fall with leaning of the motorcycle ‘‘intentional manoeuvre’’ and fall in a roundabout [2].

For each scenario, acceleration to reach the target speed of 90 kph was performed within 200 m. A flash lamp installed on the motorcycle was triggered and recorded by high-speed cameras (1000 pictures per second) for offline synchronization between video and vehicle sensor data, 5 m before the beginning of the fall area, see Fig. 2.

The high-speed cameras were also used to get the time of the fall. In Table 1, the different performed scenarios with the corresponding fall start time and the fall time for each scenario are given.

In Fig. 3, a video footage of a fall scenario is given:

The motorcycle takes run up for a distance of 200 m (not presented).

1. Five meters before the beginning of the fall area the flash lamp is triggered and recorded on the video-loggers, and the corresponding time is stamped in the data-logger.
2. The stuntman initiates the fall when he arrives to the line indicating the beginning of the fall area. The differences between the different fall scenarios are in the way stuntman initiates the fall (front or rear breaking).
3. The motorcycle starts falling, the fall start time corresponds to the time where the lowest crash protector pad hits the ground.
4. The stuntman is on the floor, this corresponds to the fall end, this fall end corresponds to the time where the stuntman's hips hits the floor.

## 4 Extreme manoeuvres design

Another data collection has been made under different extreme riding situations, such as Zigzags, extreme braking, accelerating manoeuvres, and fall-like manoeuvres. These extreme manoeuvres were conducted on track by professional riders, see Fig. 4. The aim of these experiments was to obtain a dataset describing the limit handling behaviour.
