# Supplementary material for: Dataset on powered two wheelers fall and critical events detection
Source: Data Brief. 2019 Mar 16;23:103828. doi: 10.1016/j.dib.2019.103828 (PMC6660605; doi:10.1016/j.dib.2019.103828)
Supplement: Supplementary file 1 — Multimedia component 1 [file mmc1.docx]

Conflict of Interest and Authorship Conformation Form

Please check the following as appropriate:

- All authors have participated in (a) conception and design, or analysis and interpretation of the data; (b) drafting the article or revising it critically for important intellectual content; and (c) approval of the final version.
- This manuscript has not been submitted to, nor is under review at, another journal or other publishing venue.
- The authors have no affiliation with any organization with a direct or indirect financial interest in the subject matter discussed in the manuscript
- The following authors have affiliations with organizations with direct or indirect financial interest in the subject matter discussed in the manuscript:

Author’s name Affiliation

Abderrahmane Boubezoul University Paris-Est, IFSTTAR

Fabien Dufour TROPHY R&D

Samir Bouaziz University Paris-Sud, SATIE Laboratory

Bruno Larnaudie University Paris-Sud, SATIE Laboratory

Stéphane Espié University Paris-Est, IFSTTAR
